# FAM83B inhibits ovarian cancer cisplatin resistance through inhibiting Wnt pathway

**DOI:** 10.1038/s41389-020-00301-y

**Published:** 2021-01-09

**Authors:** Shanyang He, Wei Wang, Zhiyong Wan, Hongwei Shen, Yunhe Zhao, Zeshan You, Jun Liu, Liwen Zhu

**Affiliations:** 1 Department of Obstetrics and Gynecology, Guangdong Provincial People’s Hospital & Guangdong Academy of Medical Sciences, 510080 Guangzhou, Guangdong China; 2 The Second School of Clinical Medicine, Southern Medical University, 510599 Guangzhou, Guangdong China; 3 Department of Obstetrics and Gynecology, The First Affiliated Hospital, Sun Yat-sen University, 510080 Guangzhou, Guangdong China

**Keywords:** Ovarian cancer, Cancer therapeutic resistance

## Abstract

Cisplatin resistance is frequently occurred in ovarian cancer therapy, understanding its regulatory mechanisms is critical for developing novel treatment methods and drugs. Here, we found ovarian cancer patients with low FAM83B levels had shorter survival time, tissues with cisplatin resistance also had low FAM83B levels, suggesting FAM83B might inhibit cisplatin resistance. FAM83B overexpression inhibits cisplatin resistance showed in increased ovarian cancer cell proliferation and growth rate, and reduced apoptosis rate, while FAM83B knockdown promotes cisplatin resistance. Mechanism analysis showed FAM83B interacted with APC to inhibit Wnt pathway activity, causing ovarian cancer cisplatin resistance. We also found FAM83B levels were negative with Wnt pathway activity in clinic samples, confirming FAM83B inhibited Wnt pathway activity. In summary, we found FAM83B inhibits ovarian cancer cisplatin resistance through inhibiting Wnt pathway, providing a new target for ovarian cancer therapy.

## Background

Ovarian cancer is the most common cause of gynecological-cancer-associated death. Ovarian cancer cells frequently generate apoptosis-inducing chemotherapy resistance, about over half of the women diagnosed this disease generate chemotherapy resistance and relapse^1^. Cisplatin is anti-tumor agent and generally used for various tumor treatment, it can activate signaling pathway involved in regulation of cell cycle, apoptosis, and DNA damage repair^1,2^. Cisplatin is one of the most actively used drugs for ovarian cancer treatment, but the resistance is easily generated during patients’ treatment^3^. So, it’s important to understand the regulatory mechanism of cisplatin resistance generation.

Many studies show Family with sequence similarity 83 member B (FAM83B) is an oncogene, and promotes cell transformation through activating various pathway in different kinds of tumors^4^. For example, FAM83B promotes endometrial cancer cell proliferation and metastasis through activating PI3K/AKT/mTOR and MAPK pathway^5–7^. FAM83B is a poor prognostic factor for pancreatic ductal adenocarcinoma (PDAC) and promotes PDAC proliferation^8^. FAM83B is a diagnosis and progression biomarker for lung squamous cell carcinoma^9,10^. Long non-coding RNA LINC00324 promotes gastric cancer proliferation through binding HuR and stabling FAM83B expression^11^. However, role of FAM83B in ovarian cancer cisplatin resistance has not been studied. In this study, we found ovarian cancer cells with high FAM38B levels had low cisplatin resistance, mechanism analysis showed FAM83B inhibits cisplatin resistance of ovarian cancer through interacting with APC and inhibiting Wnt pathway.

## Materials and methods

### Cell culture and clinic specimens

Ovarian cancer cell lines COV362 and SK-OV-3 were purchased from the Cell Bank of the Chinese Academy of Science (Shanghai) and maintained in high-glucose Dulbecco’s modified Eagle medium (Hyclone) supplemented with 10% fetal bovine serum (Thermo). Cells were incubated at 37 °C in a humidified atmosphere containing 5% CO_2_. The cells were identified by STR. All cell lines tested negative for mycoplasma.

Eight fresh ovarian cancer tissues were obtained from the First Affiliated Hospital, Sun Yat-sen University. Samples were snap frozen immediately and stored at −80 °C. The paraffin-embedded ovarian cancer tissues were collected from 268 women with primary epithelial ovarian cancer, who had undergone initial surgery at the First Affiliated Hospital, Sun Yat-sen University. The detailed information was shown in Supplemented Table 1. For the research proposes using these clinic samples, prior patient’s consent and approval from the Institutional Research Ethics Committee of the First Affiliated Hospital, Sun Yat-sen University.

### Immunohistochemistry (IHC)

The ovarian cancer tissue slides were deparaffinized, rehydrated, and placed into citric acid buffer for heating for 10 min. The endogenous peroxidase activity was blocked by incubating with 3% H_2_O_2_ for 10 min. Then, sections were incubated with blocking buffer for 1 h and then incubated overnight at 4 °C with anti-FAM83B antibody (1:50, PA5-56754, Thermo). Following a 10-min incubation of biotinylated second antibody, the slides were again incubated with streptavidin-peroxidase under the same condition. The immunoreaction was then visualized by incubation with diaminobenzidine chromogen (DAB) for 5 min. Finally, the slides were counterstained with hematoxylin, dehydrated, cleared, and mounted. Scores representing the proportion of positively stained ovarian tumor cells were graded as: 0 (<10%), 1 (10–40%), 2 (40–70%), 3 (>70%). The intensity of staining was determined as: 0 (no staining), 1 (light yellow), 2 (yellow brown), 3 (brown). The staining index (SI) was calculated as the product of percentage of positive cells × staining intensity. Cutoff values for high and low expression of FAM83B were chosen based on a measurement of heterogeneity using the log-rank test with respect to overall survival. The SI score of ≤3, which was considered to be high expression, and <3, which was considered to be low expression.

### Vector construction an infection

To overexpress FAM83B, the CDS sequence of FAM83B was amplified using PCR from 293T cDNA, and subsequently subcloned into lentiviral vector pSin-EF2-Puro. To knock down FAM83B, we cloned two shRNAs into lentiviral vector PLKO.1-Puro. Viruses were packed in 293T cells using lipofectamine 3000 (Thermo) according to the instructions of manufacturer. Virus supernatants were collected at 24 and 48 h after transfection. Virus supernatants infected cells for overnight with 4 µg/ml polybrene (Sigma). The stable cell lines were screened using puromycin (Selleck).

### Western blot and quantitative real-time RT-PCR (Q-PCR)

Cell lysates were prepared using RIPA buffer (Millipore) and separated on 10% sodium dodecyl sulfate polyacrylamide gel electrophoresis gel. Nuclear proteins were isolated using Membrane and Cytosol Protein Extraction Kit (Beyotime Biotechnology). The following primary antibodies were used: anti-FAM83B (1:1000, PA5-56754, Thermo), c-myc (1:1000, #18583, CST), cyclin D1 (1:1000, #18583, CST), BCL2 (1:1000, #4223, CST), Cleaved Caspase-3 (1:1000, #9661, CST), β-catenin (1:1000, #8480, CST), Flag (1:1000, #14793, CST) and His (1:1000, #12698, CST) antibodies. Anti-GAPDH antibody (1:1000, #5174, CST) was used as the loading control for total proteins, anti-EF-1α antibody (1:1000, 05-235, Millipore) was used as the loading control for nuclear proteins.

Total RNA was isolated using TRIzol regent (Thermo) and was reversely transcribed with HiScript Reverse Transcriptase (Vazyme) according to the manufacturer’s instructions. Q-PCR was carried out with using SYBR® *Premix Ex Taq*™ II (Tli RNaseH Plus) (TaKaRa) according to the manufacturer’s instructions on a CFX96 Touch Real-time PCR Detection system (Bio-Rad). GAPDH was used for the normalization of the Q-PCR.

### Cell viability assay and apoptosis

Cell viability assay was measured using MTT. MTT was performed according to previous reports^12^. Annexin V/PI staining Kit (BD) was used to analyze apoptosis according to the manufacturer’s protocol. Briefly, 1 × 10^5^ cells were suspended in 100 µl buffer, 5 µl FITC Annexin V and 5 µl PI were added, and incubated for 15 min at room temperature. After incubation, cells were analyzed by flow cytometry. TUNEL assay was also used to determine apoptosis and was performed according to the previous reports^13^.

### TCF/LEF transcriptional activity

TCF/LEF transcriptional activity was measured using a Dual Luciferase Reporter Assay. Briefly, the reporter plasmids containing wild-type (CCTTTGATC; TOPflash, plasmid 16558) or mutated (CCTTTGGCC; FOPflash, plasmid 16559) TCF/LEF DNA binding sites were purchased from Addgene^14^, and co-transfected with pRL-TK Renilla plasmid into cells using Lipofectamine 3000 (Thermo), respectively. Forty-eight hours after transfection, luciferase activity was analyzed using the Dual-Glo Luciferase Assay Kit (Promega) according to the manufacturer’s protocol. Experiments were performed in triplicates.

### Coimmunoprecipitation (co-IP)

The full length of CDS of APC with His tag was subcloned into pcDNA 3.1 vector (Thermo), the full length of CDS of FAM83B with Flag tag was subcloned into pcDNA 3.1/CAT. Two vectors were co-transfected into COV362 cells using Lipofectamine 3000 (Thermo). Co-IP were performed according to the previous reports^15^.

### Animal model

Six to 8-weeks female nude mice were purchased from Model Animal Research Center of Nanjing University, 5 × 10^6^ SK-OV-3 with FAM83B overexpression or knockdown were resuspended in 200 µl 1% Matrigel (BD) and injected in left gluteal. Five mice for each group. After tumor size reached to about 0.5 × 0.5 cm, cisplatin was injected per week (5 mg/kg) in abdominal cavity, after 3 weeks, animals were sacrificed and tumor weights were measured. All the methods were carried out in accordance with the approved guidelines by Laboratory Animal Research Center of First Affiliated Hospital, Sun Yat-sen University, and approved by the ethics committee.

### Statistical analysis

All statistical analyses were performed using SPSS 19.0 software (IBM). A paired Student’s *t*-test, *χ*^2^ test, or Wilcoxon test were used to estimate the significance of differences between two groups. Kaplan–Meier method with log-rank test was used to calculate the progression-free survival, overall survival, and post progression survival. GSEA was performed using an online algorithm (https://www.gsea-msigdb.org/gsea/index.jsp).

## Results

### Low FAM83B level is associated with poor survival and cisplatin resistance of ovarian cancer

**Fig. 1:**
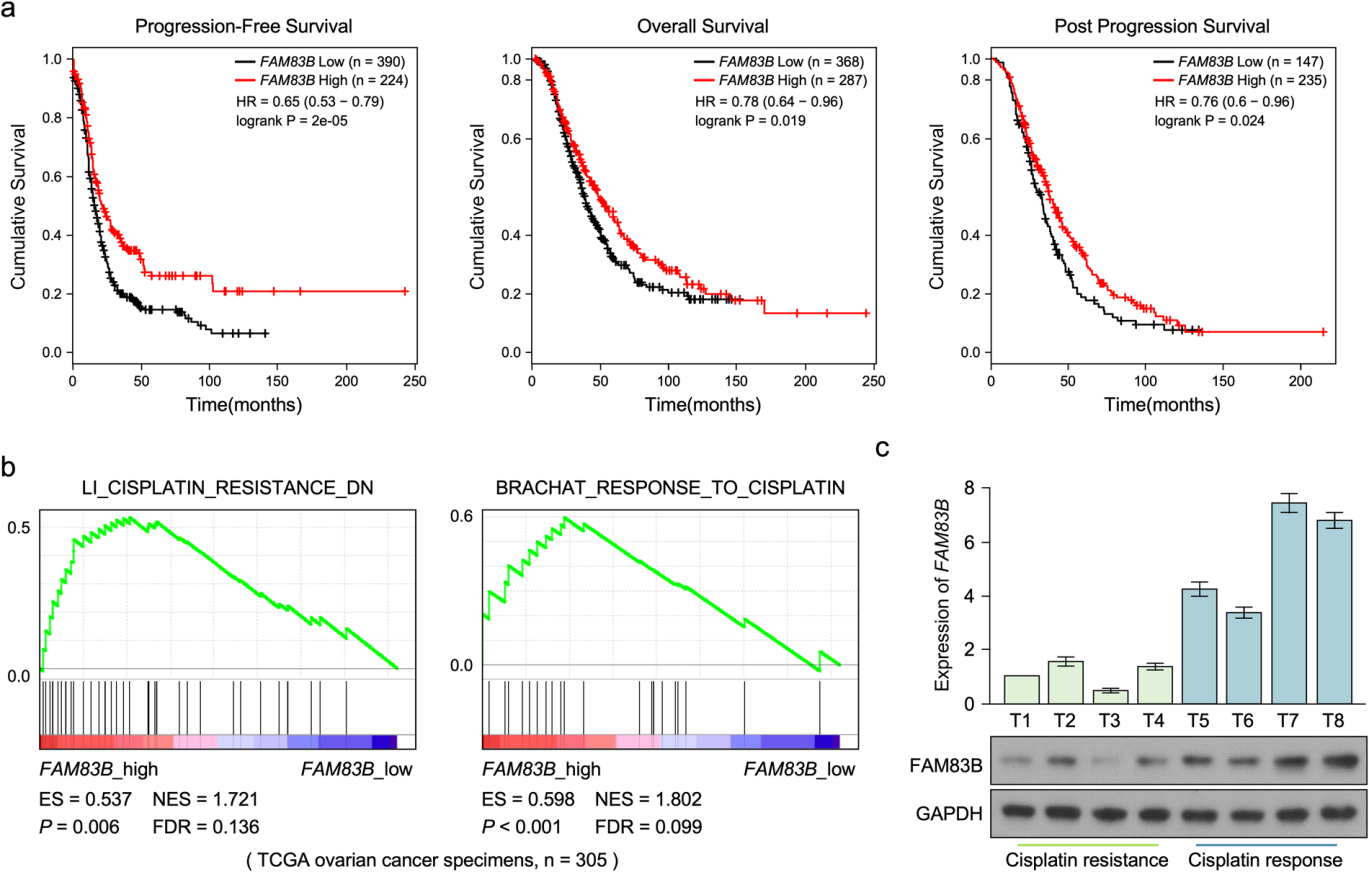
Ovarian cancer patients with low FAM83B expression had poor clinic outcome. **a** Kaplan–Meier plots indicate the progression-free survival, overall survival and post progression survival for ovarian cancer patients categorized by FAM83B expression. *P*-value is determined by log-rank test. **b** GSEA analysis of the relationship between FAM83B level and cisplatin resistance. **c** Q-PCR and western blot analysis of FAM83B expression in ovarian cancer tissues with cisplatin resistance and cisplatin response. *P < 0.05. Error bars in panels are defined as s.d.

To determine the role of FAM83B in ovarian cancer progression, low FAM83B expression was associated with poor progression-free survival, overall survival, and post progression survival of patients with ovarian cancer in Kaplan-Meier Plotter cohort (Fig. 1a). GSEA assay showed that high FAM83B levels were significantly negative with cisplatin resistance generation, while low FM83B levels were significantly positive with cisplatin resistance generation (Fig. 1b). We also collected eight fresh ovarian cancer tissues, four with cisplatin resistance, four with cisplatin response, Q-PCR and western blot assay showed FAM83B was significantly upregulated in cisplatin response ovarian cancer tissues, while FAM83B was downregulated in cisplatin resistance ovarian cancer tissues. These results showed low FAM83B was associated with cisplatin resistance.

**Fig. 2:**
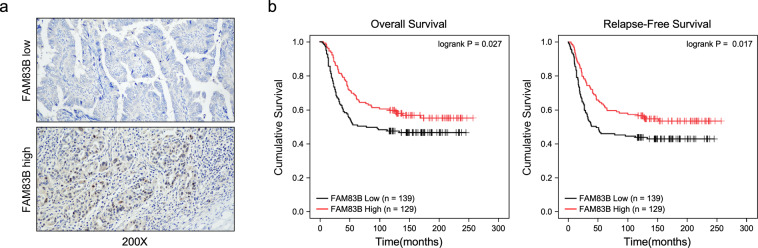
Patients with low FAM83B expression had poor clinic outcome. **a** Representative IHC pictures for FAM83B expression in ovarian cancer tissues. **b** Kaplan–Meier plots indicate the overall survival and relapse-free survival for ovarian cancer patients categorized by FAM83B expression. *p*-value is determined by log-rank test.

To confirm the above conference, we used a cohort of 268 ovarian cancer tissues to determine the relationship between FAM83B expression and survival time, IHC was used to analyze FAM83B expression of ovarian cancer tissues (Fig. 2a), We also determined the relationship between FAM83B and clinicopathologic characteristics of ovarian cancer, and found that FAM83B expression was negative with FIGO stage, metastasis, and tumor grade, while there was no relationship between FAM83B and age, menopause, relapse and status (Supplemental Table 2). Spearman correlation analysis also showed FAM83B was negatively correlated with FIGO stage, metastasis, and tumor grade (Supplemental Table 3). Kaplan–Meier survival analysis showed low FAM83B expression was associated with overall survival (*p* = 0.027) and relapse-free survival (*p* = 0.017) of patients with ovarian cancer (Fig. 2b). We used Cox regression proportional hazard analysis to determine whether FAM83B could serve as a risk factor with clinical usefulness. Multivariate analysis showed found low FAM83B expression, relapse, FIGO stage and metastasis were independent factors for ovarian cancer patients (Supplemental Table 4). These results also suggested low FAM83B was associated with poor survival, and was an independent factor for ovarian cancer patients.

### FAM83B inhibits cisplatin resistance of ovarian cancer in vitro and in vivo

**Fig. 3:**
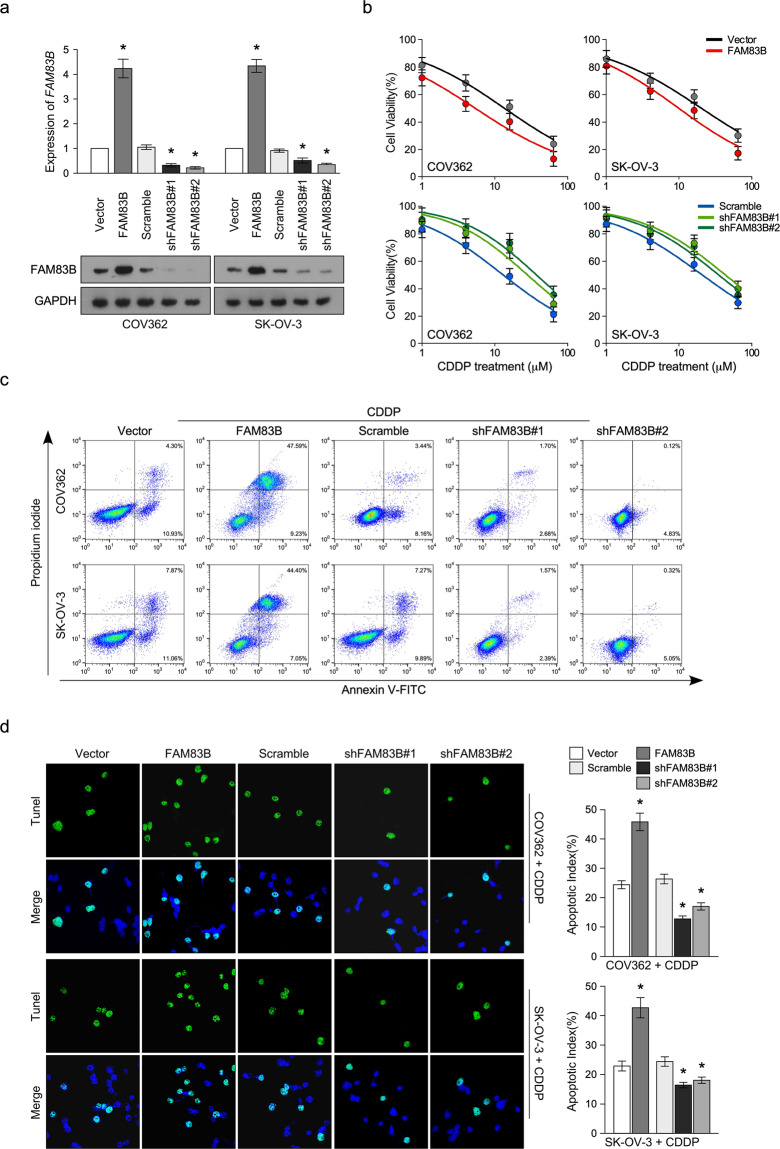
FAM83B inhibits cisplatin resistance in vitro. **a** Q-PCR and western blot analysis of FAM83B expression after infecting virus carried FAM83B overexpression vector and FAM83B shRNA vector. **b** Cell viability analysis of the effect of FAM83B overexpression or knockdown on cisplatin resistance. **c** Annexin/PI apoptosis analysis of the effect of FAM83B overexpression or knockdown on cisplatin resistance. **d** TUNEL analysis of the effect of FAM83B overexpression or knockdown on cisplatin resistance. *p < 0.05. Error bars in panels are defined as s.d.

To determine whether FAM83B regulated cisplatin resistance of ovarian cancer, we overexpressed FAM83B in ovarian cancer cell line COV362 and SK-OV-3, and knocked down FAM83B in the same cells, Q-PCR and western blot were used to determine the FAM83B expression in cells with FAM83B overexpression and knockdown (Fig. 3a). MTT assay showed FAM83B overexpression significantly inhibited ovarian cancer cell proliferation and cisplatin resistance, while FAM83B knockdown significantly increased ovarian cancer cell proliferation and cisplatin resistance (Fig. 3b). Annexin V/PI apoptotic assay showed FAM83B overexpression significantly promoted cisplatin-induced apoptosis, while FAM83B knockdown significantly inhibited cisplatin-induced apoptosis (Fig. 3c). TUNEL assay showed FAM83B overexpression significantly increased cisplatin-induced apoptosis, while FAM83B knockdown significantly inhibited cisplatin-induced apoptosis (Fig. 3d). These results showed low FAM83B level induced cisplatin resistance.

**Fig. 4:**
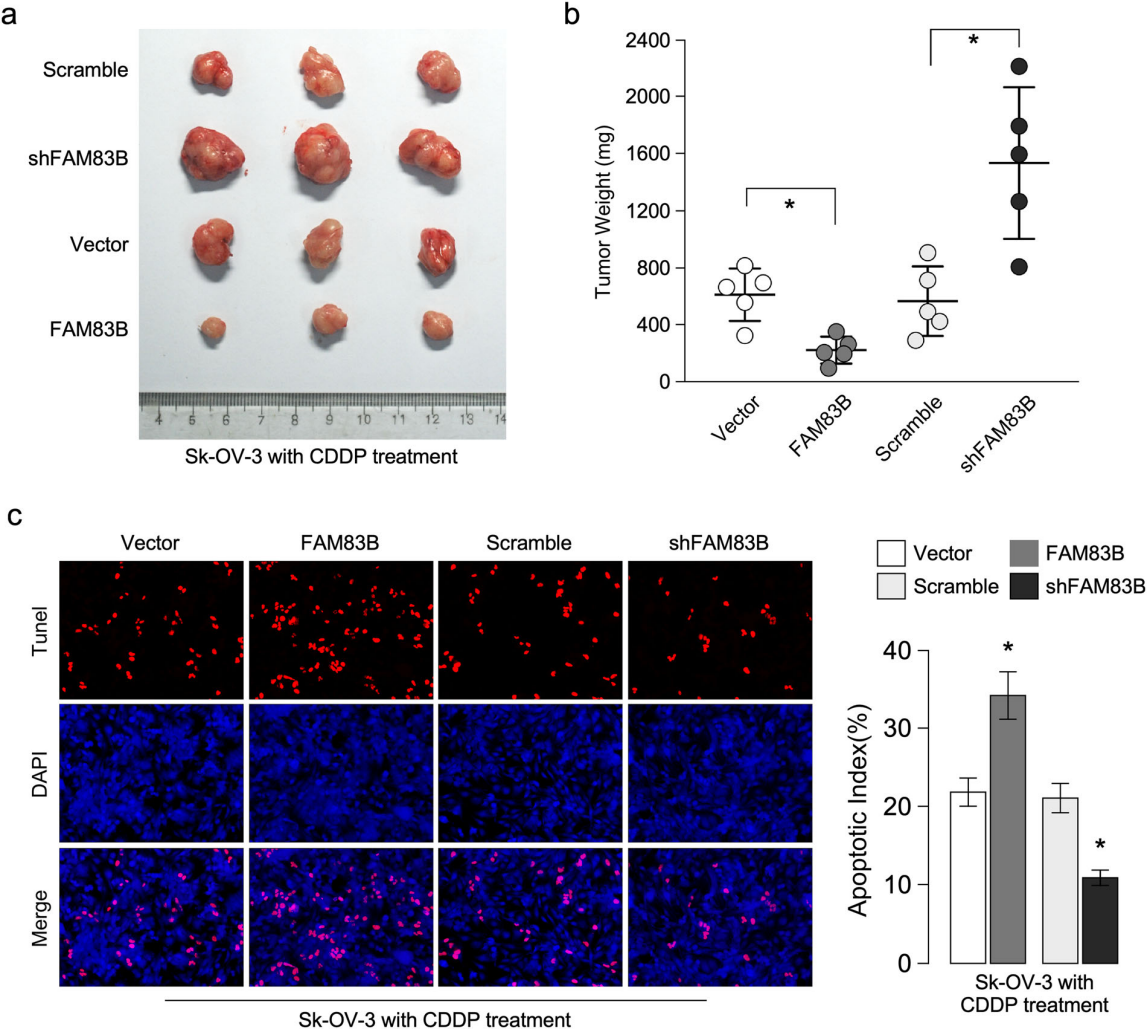
FAM83B inhibits cisplatin resistance in vivo. **a** Photographs of dissected tumors from nude mice transplanted with SK-OV-3 with FAM83B overexpression or knockdown, mice were treated with cisplatin when tumor volume reached at size reached to about 0.5 × 0.5 cm. **b** Weight of tumors from nude mice transplanted with SK-OV-3 with FAM83B overexpression or knockdown, mice were treated with cisplatin when tumor volume reached at size reached to about 0.5 × 0.5 cm. **c** TUNEL assay of apoptosis in tumor xenograft with FAM83B overexpression or knockdown. *P < 0.05. Error bars in panels are defined as s.d.

To confirm the role of FAM83B in cisplatin resistance, we determined whether FAM83B regulated cisplatin resistance in vivo *using* Sk-OV-3 cell, animal model showed tumor with FAM83B overexpression inhibited tumor growth after cisplatin treatment, while FAM83B knockdown promoted tumor growth after cisplatin treatment (Fig. 4a). Tumor weight assay also showed FAM83B overexpression significantly inhibited tumor growth after cisplatin treatment, while FAM83B knockdown significantly increased tumor growth after cisplatin treatment (Fig. 4b). We also determined cell apoptosis in Sk-OV-3 tumor with FAM83B overexpression or knockdown, TUNEL assay showed FAM83B overexpression promoted cisplatin-induced apoptosis, while FAM83B knockdown significantly inhibited cisplatin-induced apoptosis (Fig. 4c). These results showed FAM83B inhibited cisplatin resistance.

### FAM83B inhibits Wnt pathway

**Fig. 5:**
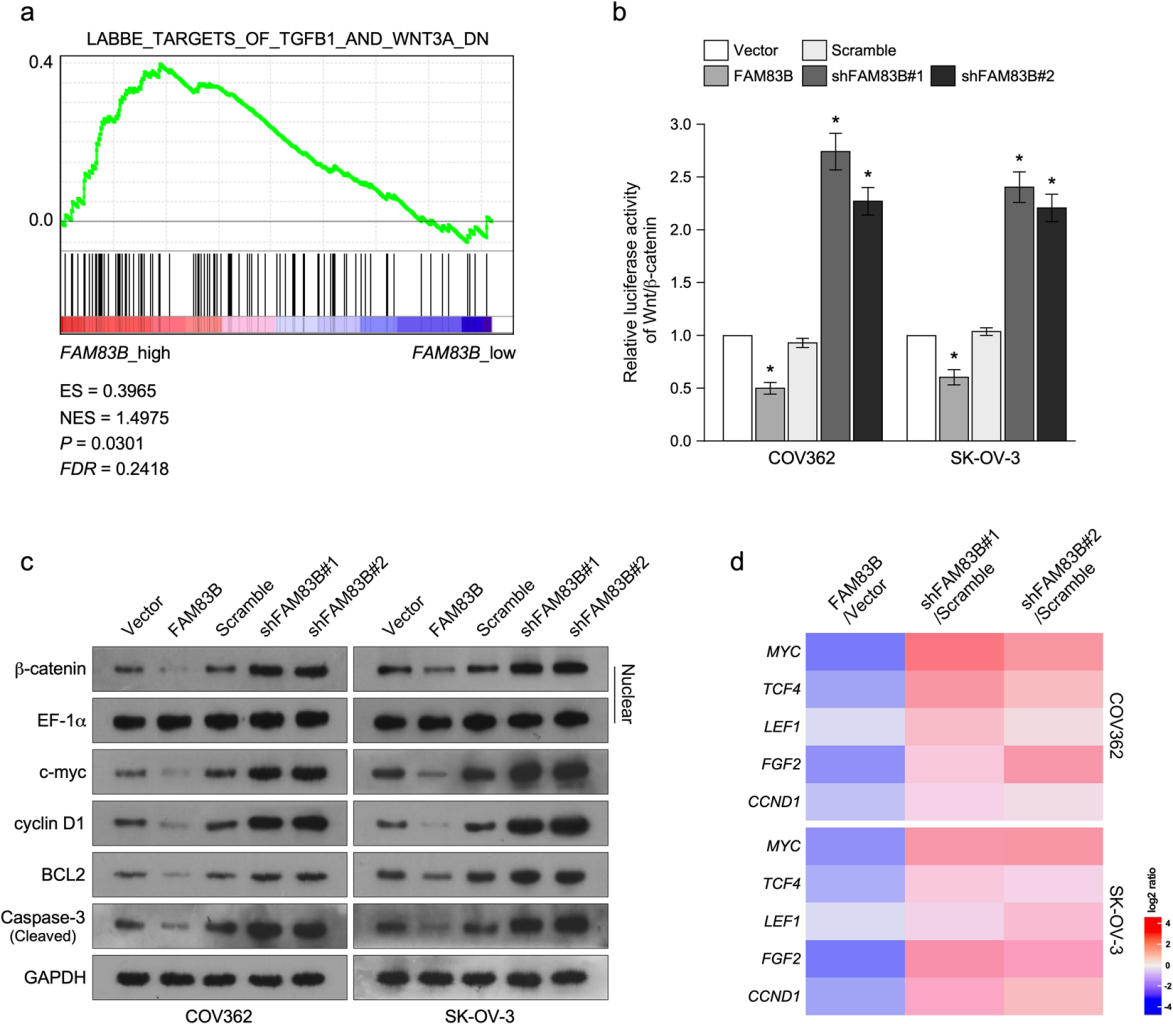
FAM83B inhibits Wnt signaling pathway. **a** GSEA analysis of the relationship between FAM83B levels and Wnt pathway. **b** Luciferase reporter assay of the effect of FAM83B expression on Wnt pathway activity. **c** Western blot analysis of nuclear β-catenin, c-myc, cyclin D1, BCL2, and cleaved Caspase-3 expression in ovarian cancer cells with FAM83B overexpression or knockdown. **d** Q-PCR analysis of MYC, TCF4, LEF1, FGF2, and CCND1 expression in ovarian cancer cells with FAM83B overexpression or knockdown. *p < 0.05. Error bars in panels are defined as s.d.

To determine the molecular mechanism of FAM83B inhibiting Wnt pathway, we used GSEA to screen which pathway was regulated by FAM83B and found that FAM83B expression was significantly in negative correlation with TGF-β and Wnt pathway (Fig. 5a). Wnt pathway have been shown to regulate cisplatin resistance of ovarian cancer, hence we chose Wnt pathway for further study. Luciferase reporter assay showed FAM83B overexpression significantly inhibited luciferase activity, while FAM83B knockdown significantly promoted luciferase activity, suggesting FAM83B inhibited Wnt pathway (Fig. 5b). Western blot assay showed FAM83B overexpression inhibited the nuclear translocation of β-catenin, while FAM83B knockdown promoted the nuclear translocation of β-catenin, also suggesting FAM83B inhibiting Wnt pathway (Fig. 5c). We also analyzed the effect of FAM83B expression on cell proliferation and apoptosis-associated genes, such as c-myc, cyclin D1, BCL2, and cleaved Caspase-3. Western blot assay showed FAM83B overexpression inhibited their expression, while FAM83B knockdown promoted their expression, suggesting FAM83B inhibited cell proliferation and apoptosis (Fig. 5c). Q-PCR analysis also showed FAM83B overexpression inhibited MYC, TCF4, LEF1, FGF2 and CCND1, while FAM83B knockdown increased their expression (Fig. 5d). These results suggested FAM83B inhibited Wnt pathway.

### FAM83B inhibits Wnt pathway through interacting with APC

**Fig. 6:**
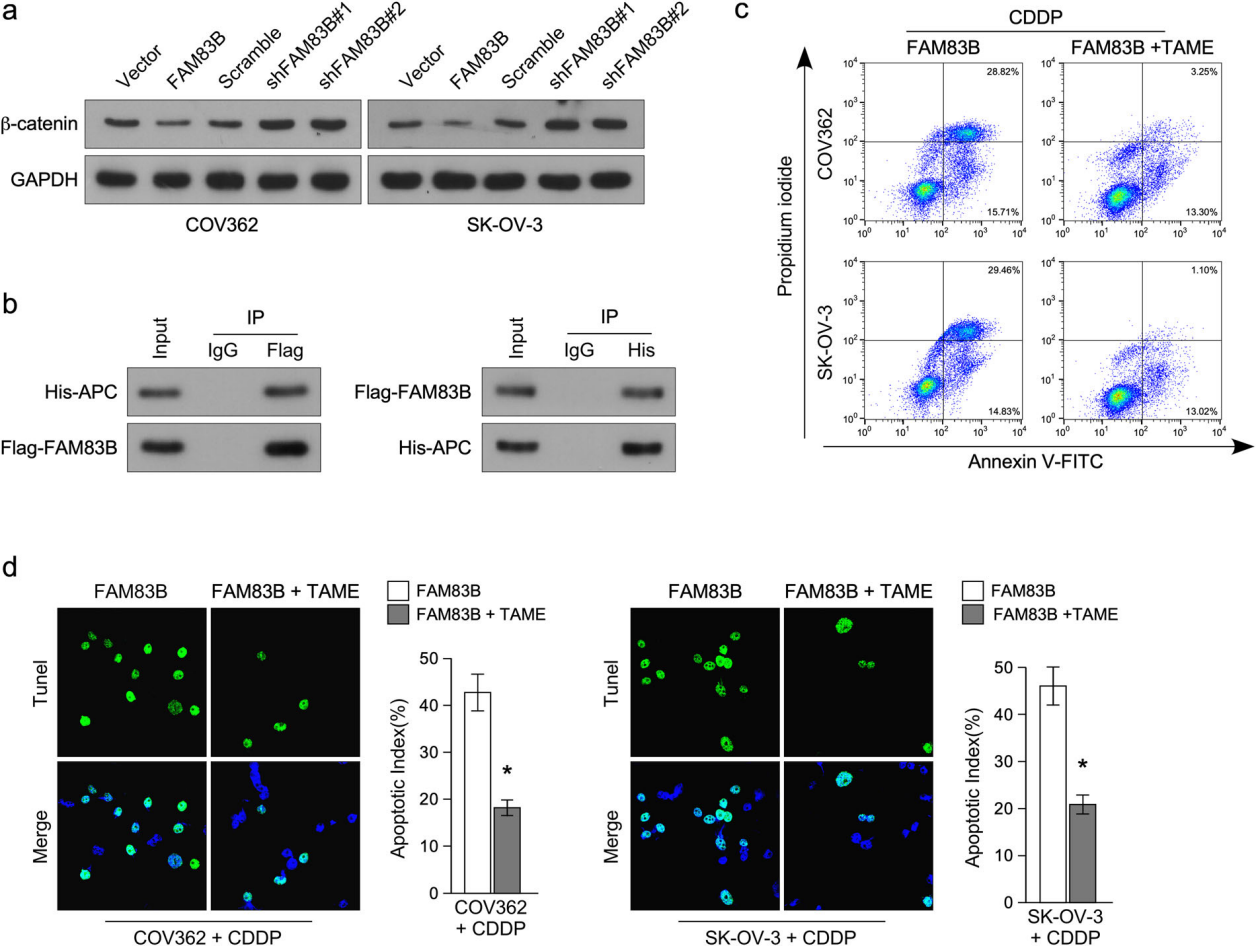
FAM83B interacts with APC. **a** Western blot analysis of β-catenin expression in ovarian cancer cells with FAM83B overexpression or knockdown. **b** IP analysis of FAM83B interacting proteins. **c** Annexin V/PI analysis of the effect of APC inhibition on cisplatin resistance in FAM83B overexpression ovarian cancer cells. **d** TUNEL analysis of the effect of APC inhibition on cisplatin resistance in FAM83B overexpression ovarian cancer cells. *p < 0.05. Error bars in panels are defined as s.d.

To determine whether FAM83B inhibits cisplatin resistance through inhibiting Wnt pathway, we used western blot to analyze total β-catenin expression and found that FAM83B overexpression inhibited β-catenin expression, while FAM83B knockdown increased β-catenin expression (Fig. 6a), suggesting β-catenin degradation was inhibited. APC protects β-catenin degradation in cytoplasm^17^, we conferred that FAM83B could competitively interact with APC causing β-catenin degradation. IP assay showed that FAM83B interacted with APC (Fig. 6b), confirming our conference. We used APC suppressor TAME to inhibit APC expression in FAM83B overexpression ovarian cancer cells^18^, Annexin V/PI assay showed APC inhibition inhibited cisplatin-induced apoptosis (Fig. 6c). TUNEL assay also showed APC inhibition inhibited cisplatin-induced apoptosis (Fig. 6d). These results suggested FAM83B inhibited Wnt pathway through interacting with APC.

**Fig. 7:**
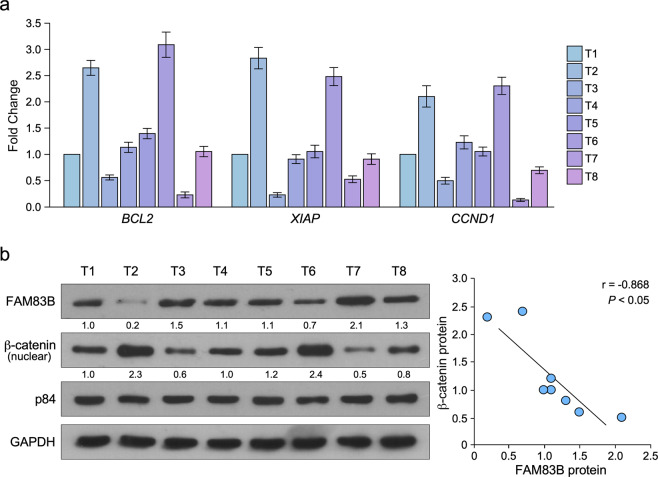
FAM83B levels were negatively correlated with Wnt pathway activity. **a** Q-PCR analysis of BCL2, XIAP and CCND1 in ovarian cancer tissues. **b** western blot analysis of FAM83B and nuclear β-catenin expression in ovarian cancer tissues. statistical analysis of the relationship between FAM83 expression and nuclear β-catenin expression in ovarian cancer tissues. Error bars in panels are defined as s.d.

We also confirmed these results using clinic samples, Q-PCR was used to analyze apoptosis and proliferation associated with genes BCL2, XIAP, and CCND1 (Fig. 7a). Western blot was used to analyze FAM83B and nuclear β-catenin expression (Fig. 7b). We found tumor tissues with high FAM83B expression had high BCL2 and CCND1 expression, and had low apoptosis inhibitor XIAP expression. Statistical analysis showed FAMB3B expression was negatively correlated with nuclear β-catenin expression (Fig. 7b). These results showed FAM83B inhibits Wnt pathway in clinic samples.

## Discussion

In present study, we found ovarian cancer patients with low FAM83B expression had poor prognosis, tissues with low FAM83B had cisplatin resistance, suggesting FAM83B might regulate ovarian cancer cisplatin resistance. Functional assay suggested FAM83B overexpression inhibited ovarian cancer cisplatin resistance, while FAM83B knockdown inhibited ovarian cancer cisplatin resistance. Mechanism analysis showed FAM83B inhibited Wnt pathway through interacting with APC. Inhibition of APC in FAM83B overexpression ovarian cancer cell increased cisplatin resistance. In ovarian cancer clinic samples, FAM83B levels were negatively correlated with Wnt pathway activity and apoptosis, confirming that FAM83B was negatively correlated with Wnt pathway activity.

β-catenin is the transcriptional coactivator of Wnt pathway, in the absence of extracellular Wnt stimulus, cytoplasmic β-catenin is degraded by destruction complex, the destruction complex is assembled by β-catenin, GSK3, CK1, Axin, APC, and β-TrCP. GSK3 and CK1 phosphorylates β-catenin, and β-TrCP is a F-box containing E3-ligase and interacts with phosphorylated β-catenin to degrade β-catenin. While Wnt ligands bind to receptor, β-catenin is uncoupled from the destruction complex and translocate to the nucleus, β-catenin binds to TCF/LEF to activate Wnt target gene transcription in nucleus^19–22^. We found FAM83B overexpression inhibits nuclear translocation of β-catenin, while FAM83B inhibition knockdown increased nuclear translocation of β-catenin. Luciferase reporter assay showed FAM83B overexpression inhibited TCF/LEF transcriptional activity, while FAM83B knockdown promoted TCF/LEF transcriptional activity, these results suggested FAM83 inhibited Wnt pathway. c-myc, cyclin D, BCL2, and caspase-3 are the targets of Wnt pathway, c-myc has been showed to regulate ovarian cancer cisplatin resistance, cyclin D is associated with cell proliferation^23^, BCL2 and caspase-3 are apoptosis inhibitors^24^. These results also suggested FAM83B inhibits ovarian cancer cisplatin resistance.

APC binds to β-catenin and Axin and could serve as a scaffold for destruction complex^16^, we found FAM83B interacts with APC, suggesting FAM83B might stabilize the destruction complex to inhibit Wnt pathway activity, but detailed mechanism must be further studied. We inhibited APC using small molecule compound in FAM83B overexpression ovarian cancer cells and found cisplatin resistance was increased, suggesting FAM83B inhibited cisplatin resistance through interacting with APC to inhibit Wnt pathway. Finally, we found FAM83B was negatively correlated with β-catenin expression in nucleus, confirming FAM83B inhibited Wnt pathway activity.

## Conclusion

In summary, we found FAM83B inhibits ovarian cancer cisplatin resistance through interacting with APC to suppress Wnt pathway activity, and provides a target for ovarian cancer therapy.

## Supplementary information

Supplemental table 1

Supplemental table 2

Supplemental table 3

Supplemental table 4
