# Correction to: FAM83B inhibits ovarian cancer cisplatin resistance through inhibiting Wnt pathway

**DOI:** 10.1038/s41389-021-00303-4

**Published:** 2021-02-10

**Authors:** Shanyang He, Wei Wang, Zhiyong Wan, Hongwei Shen, Yunhe Zhao, Zeshan You, Jun Liu, Liwen Zhu

**Affiliations:** 1 Department of Obstetrics and Gynecology, Guangdong Provincial People’s Hospital and Guangdong Academy of Medical Sciences, 510080 Guangzhou, Guangdong China; 2 The Second School of Clinical Medicine, Southern Medical University, 510599 Guangzhou, Guangdong China; 3 Department of Obstetrics and Gynecology, The First Affiliated Hospital, Sun Yat-sen University, 510080 Guangzhou, Guangdong China

**Keywords:** Ovarian cancer, Cancer therapeutic resistance

Correction to: *Oncogenesis*

10.1038/s41389-020-00301-y published online 9 January 2021

The original version of this article unfortunately contained a mistake. The following correction has therefore been made in the original: Affiliations 1 and 3 were interchanged and affiliation 2 was incomplete. The original article has been corrected.

