# Correction: FAM83B inhibits ovarian cancer cisplatin resistance through inhibiting Wnt pathway

**DOI:** 10.1038/s41389-023-00459-1

**Published:** 2023-03-15

**Authors:** Shanyang He, Wei Wang, Zhiyong Wan, Hongwei Shen, Yunhe Zhao, Zeshan You, Jun Liu, Liwen Zhu

**Affiliations:** 1 Department of Obstetrics and Gynecology, Guangdong Provincial People’s Hospital & Guangdong Academy of Medical Sciences, 510080 Guangzhou, Guangdong China; 2 The Second School of Clinical Medicine, Southern Medical University, 510599 Guangzhou, Guangdong China; 3 Department of Obstetrics and Gynecology, The First Affiliated Hospital, Sun Yat-sen University, 510080 Guangzhou, Guangdong China

**Keywords:** Ovarian cancer, Cancer therapeutic resistance

Correction to: *Oncogenesis* 2021;10:6 10.1038/s41389-020-00301-y, published online 09 January 2021

Following the publication of this paper, the authors noted the following errors:

- In the Results section (17th line, page 3) the name of Database was listed as “TCGA”. This should be “Kaplan–Meier Plotter”.
- In the Results section (7th line, page 4), the sentence “To confirm the above conference, we used a cohort of 286 ovarian cancer tissues…” should be “To confirm the above conference, we used a cohort of 268 ovarian cancer tissues…”
- In the Materials and methods section under “Animal model”, the sentence “Three mice for each group” should be “Five mice for each group”.
- During assembly of Figure 3C, the same images were presented in error for COV362-Scramble and ASKOV3-Scramble. The correct image can be seen below.

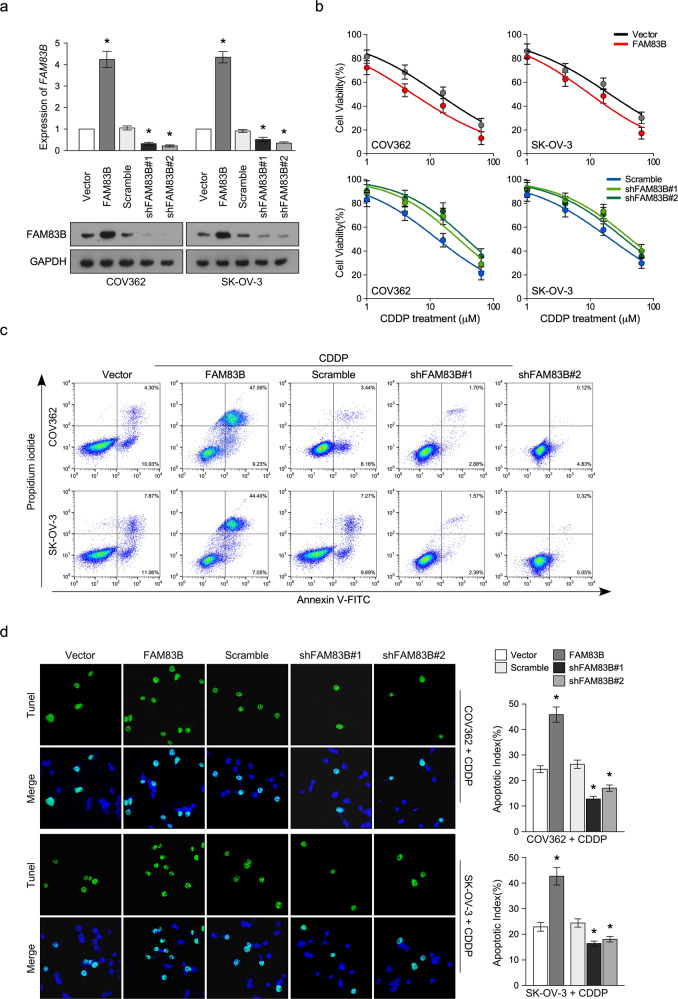


The authors confirm that these corrections have no impact on the conclusions of this article.

The original article has been corrected.

