# Supplementary material for: FAM83B inhibits ovarian cancer cisplatin resistance through inhibiting Wnt pathway
Source: Oncogenesis. 2021 Jan 9;10(1):6. doi: 10.1038/s41389-020-00301-y (PMC7797002; doi:10.1038/s41389-020-00301-y)
Supplement: Supplementary file 1 — Supplemental table 1 [file 41389_2020_301_MOESM1_ESM.docx]

**Supplemental table 1. Clinicopathological characteristics of studied patients and expression of FAM83B in ovarian cancer**

| Characteristics | No. of Cases |
| --- | --- |
| **Age (years)** |  |
| ≤55 | 179 |
| ＞55 | 89 |
| **Metastasis** |  |
| Negative | 70 |
| Positive | 198 |
| **FIGO Stage** |  |
| I &II | 102 |
| III & IV | 166 |
| **Pathological Grade** |  |
| G1 | 45 |
| G2 | 120 |
| G3 | 103 |
| **Menopause** |  |
| Yes | 139 |
| Not | 129 |
| **Relapse** |  |
| Yes | 138 |
| Not | 130 |
| **Status (at follow-up)** |  |
| Alive | 138 |
| Death because of ovarian cancer | 130 |
| Death because of other than ovarian cancer | 0 |
| **FAM83B expression** |  |
| Negative | 8 |
| Positive | 260 |
| Low expression | 139 |
| High expression | 129 |
