# Supplementary material for: FAM83B inhibits ovarian cancer cisplatin resistance through inhibiting Wnt pathway
Source: Oncogenesis. 2021 Jan 9;10(1):6. doi: 10.1038/s41389-020-00301-y (PMC7797002; doi:10.1038/s41389-020-00301-y)
Supplement: Supplementary file 2 — Supplemental table 2 [file 41389_2020_301_MOESM2_ESM.docx]

**Supplemental Table 2. Correlation between FAM83B expression and clinicopathologic characteristics of Ovarian cancer**

| **Characteristics** | | **FAM83B** | | **Chi-square test**  ***P*-value** | **Fisher’s Exact**  **test *P*-value** |
| --- | --- | --- | --- | --- | --- |
|  |  | **Low No. cases** | **High No. cases** |  |  |
| **Age** | < 55 | 89 | 90 | 0.364 | 0.364 |
|  | ≥ 55 | 50 | 39 |  |  |
| **Menopause** | Yes | 75 | 64 | 0.541 | 0.541 |
|  | No | 64 | 65 |  |  |
| **FIGO Stage** | I | 15 | 54 | 0.001 | 0.001 |
|  | II | 14 | 19 |  |  |
|  | III | 80 | 44 |  |  |
|  | IV | 30 | 12 |  |  |
| **Metastasis** | Yes | 124 | 74 | 0.001 | 0.001 |
|  | Not | 15 | 55 |  |  |
| **Relapse** | Yes | 79 | 59 | 0.087 | 0.087 |
|  | Not | 60 | 70 |  |  |
| **Tumor grade** | 1 | 10 | 35 | 0.001 | 0.001 |
|  | 2  3 | 60  69 | 60  34 |  |  |
| **Stutas** | Survive | 65 | 73 | 0.114 | 0.114 |
|  | Mortality | 74 | 56 |  |  |
