# Supplementary material for: FAM83B inhibits ovarian cancer cisplatin resistance through inhibiting Wnt pathway
Source: Oncogenesis. 2021 Jan 9;10(1):6. doi: 10.1038/s41389-020-00301-y (PMC7797002; doi:10.1038/s41389-020-00301-y)
Supplement: Supplementary file 3 — Supplemental table 3 [file 41389_2020_301_MOESM3_ESM.docx]

**Supplemental Table 3. Spearman correlation analysis between FAM83B and clinical pathologic factors**

| **Variables** | **FAM83B Expression Level** | |
| --- | --- | --- |
|  | **Spearman Correlation** | ***P*-Value** |
| **Age** | -0.061 | 0.321 |
| **Menopause** | -0.043 | 0.479 |
| **FIGO Stage** | -0.373 | 0.001 |
| **Metastasis** | -0.362 | 0.001 |
| **Relapse** | -0.111 | 0.070 |
| **Tumor Grade** | -0.297 | 0.001 |
| **Survive or Mortality** | -0.098 | 0.109 |
