# Supplementary material for: FAM83B inhibits ovarian cancer cisplatin resistance through inhibiting Wnt pathway
Source: Oncogenesis. 2021 Jan 9;10(1):6. doi: 10.1038/s41389-020-00301-y (PMC7797002; doi:10.1038/s41389-020-00301-y)
Supplement: Supplementary file 4 — Supplemental table 4 [file 41389_2020_301_MOESM4_ESM.docx]

**Supplemental Table 4. Univariate and multivariate analyses of various prognostic parameters in patients with BC Cox-regression analysis**

|  | **Multivariate analysis** | | | |  |
| --- | --- | --- | --- | --- | --- |
|  | **No. patients** | ***P*** | **Relative risk** | **95% confidence interval** | |
| **Relapse** |  |  |  |  |  |
| **Yes** | 138 | 0.001 | 222.632 | 45.904-1079.759 | |
| **Not** | 130 |  |  |  |  |
| **FIGO Stage** |  |  |  |  |  |
| **I & II** | 102 | 0.001  0.001 | 3.376  9.926 | 2.644-4.311  4.335-19.932 | |
| **III & IV** | 166 |  |  |  |  |
| **Metastasis**  **Yes**  **No** | 70  198 |  |  |  |  |
| **Expression of FAM83B** |  |  |  |  |  |
| **Low expression** | 139 | 0.028 | 0.678 | 0.479-0.960 | |
| **High expression** | 129 |  |  |  |  |
